# The perspectives of individuals with incomplete spinal cord injury on measuring the intensity of balance challenge during reactive balance training: A qualitative study

**DOI:** 10.1371/journal.pone.0352139

**Published:** 2026-06-25

**Authors:** Matthew G. Heffernan, Anna Ogonowska-Slodownik, Alyssa Benitez, Katherine Chan, Elizabeth L. Inness, Kristin E. Musselman

**Affiliations:** 1 Rehabilitation Sciences Institute, Temerty Faculty of Medicine, University of Toronto, Toronto, Ontario, Canada; 2 KITE Research Institute, Toronto Rehabilitation Institute - University Health Network, Toronto, Ontario, Canada; 3 Department of Physical Therapy, Temerty Faculty of Medicine, University of Toronto, Toronto, Ontario, Canada; SARAH Network of Rehabilitation Hospitals: Rede SARAH de Hospitais de Reabilitacao, BRAZIL

## Abstract

**Importance:**

Balance rehabilitation is an important component of recovery for individuals with incomplete spinal cord injury (iSCI), who are at heightened risk of falls due to impaired postural control. Intensity of balance challenge refers to the postural control demand of a task relative to an individual’s maximum capacity. Achieving adequate intensity of balance challenge is essential for appropriate prescription of balance exercises. Balance intensity is often assessed through self-report on an ordinal scale. However, there is limited understanding of how individuals with iSCI interpret the concept of balance intensity and use self-reported scales.

**Design:**

A qualitative descriptive study was conducted. Twelve individuals with iSCI (nine male, three female; mean age 67.2 ± 12.9 years) participated while undergoing Reactive Balance Training. Over six weeks, participants rated the balance challenge intensity of each exercise using an 11-point ordinal scale. Once per session, participants explained their ratings to a researcher who recorded the comments as field notes. After completing ≥10 training sessions, participants completed a semi-structured interview exploring their definitions of balance challenge, factors influencing their difficulty ratings, and their views on the Balance Intensity Scale, an alternative measure used with older adults. Interviews were audio recorded, transcribed verbatim, and analyzed alongside field notes using a conventional content analysis.

**Results:**

Five themes were identified: 1) Influence of task familiarity on perceived balance challenge, 2) Influence of fear of falling on perceived balance challenge, 3) Misuse of ordinal scale, 4) Ambiguity of ordinal scale, and 5) Concern of the time demand of scales. Participants are concerned about lost training time and value clinician expertise over their self-reported rating for efficient assessment.

**Conclusions:**

Participants’ perspectives on measuring balance intensity highlight the complexity of this concept. Future work could employ quantitative evaluations of the validity of self-reported balance intensity and consider the tools/skills used by clinicians.

## Introduction

Incomplete spinal cord injury (iSCI) is one of many neurological conditions that impair an individual’s balance control, leading to an increased risk of experiencing falls, which can have serious impacts on mobility and independence [1,2]. Additionally, low balance confidence and a fear of falling can compound the negative impact of impaired balance control, leading to decreases in activity levels and quality of life [3–5]. Balance ability also tends to deteriorate with age and has been a focus of researchers seeking to reduce the heightened risk of falls in the neurologically impaired and elderly populations [6–10].

Interventions like Reactive Balance Training (RBT) can effectively improve balance ability for individuals with balance impairments (i.e., SCI, stroke, multiple sclerosis, Parkinson’s disease, older adults) [11–19]. The effectiveness of balance training interventions like RBT are largely influenced by the dose delivered (i.e., frequency, intensity, time, and type), however, the connection between dose and effectiveness is poorly understood [20–24]. In particular, intensity is recognized as an important component in ensuring adequate dosage is achieved in balance training. Intensity in this context refers to the extent to which the balance control system of a given individual is challenged relative to that individual’s capacity to maintain balance [25]. Therefore, to be effective, balance training interventions must be sufficiently challenging, with exercises performed near an individual’s maximum capacity [20,21,26–29].

Despite the importance of intensity in relation to balance challenge, a significant gap exists in our understanding of how it should be assessed and reported in both research and clinical practice [30]. Many studies evaluating balance training interventions do not adequately report the intensity of the balance challenge [30]. A systematic review found that measures intended to assess balance challenge intensity often assess other types of exercise instead and do not consider the individual’s capacity to maintain balance [30]. The intensities of other types of exercise are more easily understood. For example, the intensity of aerobic exercise is quantified using measures of maximal heart rate, and the intensity of resistance exercise can be related to the individual’s 1-repetition maximum [20]. However, it is difficult to accurately quantify the intensity of balance challenge, and most clinicians rely on their professional expertise and training to make an assessment [31,32]. This involves observing the markers thought to be associated with the degree of challenge of a given exercise (i.e., pausing before starting a task, or postural reactions) [25], and adjusting the intensity of the task accordingly.

Self-report measures of intensity have also been used in balance training to support the expert assessment of clinicians. Many of these tools are similar to those used in the assessment of aerobic exercise intensity (e.g., Borg Rating of Perceived Exertion) and often involve the rating of balance challenge on an ordinal scale. One example is the Rate of Perceived Stability (RPS) scale, which has been shown to measure intensity of balance challenge independent of the exertional effort of training (i.e., increased heart rate) [33]. These scales have been used during balance exercise in individuals with stroke [34] and multiple sclerosis [35], showing promise in their ability to provide clinicians with quantitative information about their clients’ perceived balance challenge intensity. However, the subjective nature of the scales may represent a limitation, as they rely on self-perception, which is influenced by personal factors and may not accurately reflect the true intensity of a task [36–38]. Additionally, participants with balance impairments have demonstrated a tendency to underestimate the intensity of balance training exercises compared to clinician ratings [37]. The Balance Intensity Scales (BIS) (available from https://www.monash.edu/medicine/balance-intensity-scale) have recently been developed for use in older adults to assess both therapist (BIS-T) and exerciser (BIS-E) perspectives on intensity of balance challenge based on observations [39]. These scales involve both an ordinal rating of effort and an additional list of items meant to deliver a more objective assessment of balance challenge based on the biomarkers known to be associated with loss of balance [25,39]. However, the BIS-T and BIS-E have not been validated for use in individuals with SCI.

For individuals with iSCI, achieving adequate intensity is a fundamental component of balance training that leads to improvements in functional outcomes. However, the tools used to measure the intensity of balance challenge may not provide the necessary precision to tailor interventions effectively. Understanding how individuals with iSCI perceive the intensity of balance challenge and their perspectives on the use of measurement tools is essential. Their unique sensory and motor impairments may influence their perception of balance and the intensity of balance exercises. Exploring their definitions of balance challenge, the factors they consider when rating difficulty and their views on the utility of different assessment methods can provide valuable insights for improving the prescription and evaluation of balance training interventions in this population. This study aims to understand the perspectives of individuals with iSCI on measuring the intensity of balance challenge during RBT, including their experiences using an ordinal scale and their views on an alternative measurement tool.

## Materials and methods

A qualitative descriptive study [40,41] was conducted at the Lyndhurst Centre, Toronto Rehabilitation Institute–University Health Network, an SCI rehabilitation hospital in Canada. This study was part of a larger randomized clinical trial (ClinicalTrials.gov identifier NCT04881565) comparing clinical outcomes of RBT combined with functional electrical stimulation (FES) to RBT alone [42]. Approval was granted by University Health Network’s Research Ethics Board (study ID: 21–5210). The Standards for Reporting Qualitative Research (SRQR) were followed for study reporting [43].

### Participants

Involvement in this portion of the larger clinical trial was optional, and twelve individuals agreed to participate. Inclusion criteria for the clinical trial required participants to: 1) be ≥ 18 years old, 2) have a chronic (>1 year post-injury) traumatic or non-progressive, non-traumatic motor iSCI (i.e., American Spinal Injury Association Impairment Scale C or D), 3) ambulate 10 meters with assistance (i.e., Walking Index for SCI II rating of 6–19) [44], and 4) be able to stand for >30 seconds without upper limb support or assistance (i.e., scored ≥2/4 on the Standing Unsupported item from the Berg Balance Scale) [45]. Participants were excluded from participating in the clinical trial if they demonstrated any of the following: 1) contraindications to FES, 2) no motor response to FES, 3) other conditions affecting balance, 4) pressure injuries on the pelvis or trunk, 5) history of lower limb fragility fractures, or 6) severe spasticity or contracture in the lower limbs. Recruitment occurred via Lyndhurst Centre’s Central Recruitment Database from April 3, 2022 to March 31, 2024 [46]. Written informed consent was obtained from all participants before enrolment and confirmed before the audio recording of semi-structured interviews.

### Intervention

**Fig 1:**
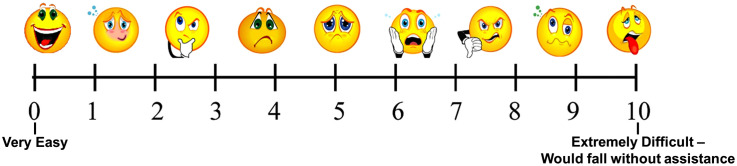
Intensity scale for reactive balance training. 11-point ordinal scale used by individuals with motor incomplete spinal cord injury for the self-reported rating of intensity of balance challenge during Reactive Balance Training.

The clinical trial involved participants completing an intensive six-week RBT program of 18 one-hour sessions. Training was delivered by a physical therapist (KEM) or kinesiologist (MGH, KC) and involved manual perturbations applied during standing and walking tasks (e.g., side stepping, kicking a ball, weight shifting) tailored to each participant’s ability level. Participants were secured in a safety harness throughout training. After each task, participants rated the perceived intensity of balance challenge using an 11-point ordinal scale (0 = very easy, 10 = extremely difficult – would fall without assistance) (Fig 1) [11,47]. Task difficulty was adjusted to elicit ratings of ≥7.

### Data collection

At least once during each training session, participants were asked to provide verbal explanations for their intensity ratings to the clinician, who immediately documented their responses verbatim as field notes using a standardized data collection sheet. A researcher (KC) later transcribed these field notes [48]. Participants engaged in a semi-structured interview after completing ≥ 10 sessions (i.e., halfway through the training protocol), when they had become familiar with the use of the ordinal scale to rate intensity of balance challenge. Interviews were conducted in person by a male researcher and kinesiologist who had delivered RBT training sessions to the participants and had four years of qualitative research experience (MGH). A semi-structured interview guide created by MGH and KEM (a physical therapy researcher with >10 years of qualitative research experience) was followed (Table 1). MGH and KEM met following every 1–2 interviews to reflect on the interview discussions and consider possible edits to the interview guide and/or delivery. Interview questions were open-ended to explore participants’ perceptions in three areas: 1) definitions of “balance challenge”, 2) factors influencing ratings on the ordinal scale used in their training, and 3) perceptions of an alternative tool (BIS-E) involving objective measures of balance control. The BIS-E includes a 5-point ordinal scale and an objective item checklist, developed through a rigorous process to identify markers of balance challenge (i.e., bracing, postural reactions, time delay to task commencement, and pre-task talk) in older adults [39]. The objective item checklist is a novel element in balance intensity assessment. Initial assessments have demonstrated that the BIS-E is a unidimensional measure of balance intensity during exercise, and the items included in the checklist follow a hierarchical distribution [39]. Additionally, participants’ ratings on the 5-point ordinal scale correlated with therapist scores on the BIS-T [39]. Interviews were audio-recorded, transcribed verbatim using Microsoft Word (MGH, AB), and anonymized. Transcriptions of field notes and interviews were stored securely on an institutional research drive.

**Table 1:** Semi-structured interview guide.

Interview Question	Probes
Can you please describe your experience with the balance training sessions so far?	What activities or tasks do you practice?
	Have the activities/tasks changed over the course of the balance training sessions?
In the training sessions for this study, you perform a number of tasks that are designed to challenge your balance. How would you define a balance challenge?	What exercises or activities have you found challenging?
	Why do you think they are challenging?
How do you feel about the level of challenge of the balance tasks you completed during the balance training so far?	Do you think the tasks have been sufficiently challenging? Why or why not?
	What makes a task challenging with respect to balance?
	How were you able to determine when a task was not challenging?
During your balance training sessions, we used an 11-point ordinal scale where 0 was defined as ‘very easy’ and 10 was defined as ‘very challenging, would fall without assistance’. Can you describe your understanding of the 11-point ordinal scale and how it is used to rate the difficulty of the balance exercises practiced during training sessions?	Participant was shown the scale used in training.
	What factors do you consider when providing a rating on the difficulty scale during the training sessions?
How do you feel about this scale as a measure of balance intensity/challenge?	What are some of the benefits or challenges of using this scale?
	Do you feel that the difficulty scale is a useful tool for measuring the challenge of the balance exercises? Why or why not?
	Do you have any suggestions for improving the difficulty scale?
The Balance Intensity Scale (BIS) is another instrument designed to rate the level of challenge or intensity of balance tasks. I would like to review this scale with you and ask for your opinion on this alternative scale.	Researcher reviewed the BIS-E with the participant.
How do you feel about this scale as a measure of balance intensity/challenge? (referring to the Balance Intensity Scale)	Which items from the scale do you feel closely relate to balance challenge?
	Are there any items that you feel are unrelated to balance challenge? If yes, please elaborate.
	What would be some of the benefits or challenges of using this scale?
How do you feel about using this scale during balance training like the sessions from this study?	

### Data analysis

The demographic data and injury characteristics of the participants were summarized descriptively as mean ± SD or frequency counts. Field notes and interview transcripts were analyzed using conventional content analysis, an appropriate approach for investigating a research question in an area with limited existing understanding [49]. Each transcript was independently reviewed by at least two researchers (MGH, AB, AOS) who documented key ideas and recurring concepts using marginal notes. The researchers then met to discuss these ideas and resolve discrepancies in their findings through group consensus (MGH, AB, AOS, KEM). Preliminary codes were derived through these group discussions, and a codebook was developed in Microsoft Excel. The quotes from field notes and interview transcripts supporting preliminary codes were added to the codebook and condensed where appropriate to preserve core meanings. The codes were refined through repeated rounds of discussion as described above until all transcripts had been reviewed. The final codebook was applied to all transcripts by MGH. Researchers (MGH, AOS, KEM) then grouped related codes into sub-themes based on content similarity. The researchers discussed emerging themes based on the identified sub-themes and the frequency patterns of codes appearing within those sub-themes. Final themes were identified through group consensus by all authors.

## Results

Table 2 outlines the demographic and injury characteristics of the twelve participants (nine male, three female; mean age 67.2 ± 12.9 years; mean time post-injury 3.5 ± 2.2 years) who were enrolled in the balance training interventions (six participants in RBT + FES, six participants in RBT) and completed semi-structured interviews. Five themes were identified from the field notes and interview transcripts: 1) Influence of task familiarity on perceived balance challenge, 2) Influence of fear of falling on perceived balance challenge, 3) Misuse of ordinal scale, 4) Ambiguity of ordinal scale, and 5) Concern of the time demand of scales. Themes and sub-themes are included in Table 3. Additional supporting quotes are reported in a supplementary file (S1 Table).

**Table 2:** Demographic and injury characteristics of study participants.

Participant ID	Intervention Group	Gender	Age (years)	Level of Injury	Mechanism of Injury	Time Post-Injury (years)	Mobility Aid Used in Daily Life
RBT07	RBT	M	75.6	T4	Non-Traumatic	5.2	Cane
RBT10	RBT	W	51.1	C3	Non-Traumatic	6.5	Cane
RBT11	RBT + FES	M	71.3	T12	Non-Traumatic	5.0	4WW
RBT12	RBT	M	75.2	C6	Non-Traumatic	1.3	WC
RBT13	RBT + FES	M	46.0	T10	Traumatic	3.6	WC
RBT14	RBT + FES	M	66.1	C3	Traumatic	8.4	4WW
RBT15	RBT + FES	M	72.3	C4	Traumatic	2.0	Cane
RBT16	RBT	M	84.8	T9	Non-Traumatic	4.0	4WW
RBT17	RBT + FES	W	86.0	C3	Non-Traumatic	2.3	4WW
RBT18	RBT	M	71.0	C2	Traumatic	1.2	4WW
RBT19	RBT	W	49.2	C2	Traumatic	1.7	Cane
RBT20	RBT + FES	M	57.5	T3	Non-Traumatic	1.2	Cane
**Mean**			67.2			3.5	
**SD**			12.9			2.2	
**Count**	6 RBT	9 M, 3 W			5 Traumatic 7 Non-Traumatic		5 Cane, 5 4WW, 2 WC

RBT, reactive balance training; FES, functional electrical stimulation; M, man; W, woman; T, thoracic; C, cervical; 4WW, 4-wheeled walker; WC, wheelchair.

**Table 3:** Themes and sub-themes.

Themes	Sub-themes
1. Influence of task familiarity on perceived challenge	1.1 Challenges of novel tasks
	1.2 Repetition reduces perceived challenge
2. Influence of fear of falling on perceived challenge	2.1 Fear as a determinant of high intensity of balance challenge
	2.2 Emotional and physical safety considerations
3. Misuse of the Ordinal Scale	3.1 Tracking personal progress
	3.2 Comparing performance to pre-injury
	3.3 Controlling the difficulty level of tasks
4. Ambiguity of ordinal scale	4.1 The ordinal scale was confusing to use
	4.2 Challenges using the extremes of the ordinal scale
5. Concern of the time demand of scales	5.1 Training time prioritized over rating time
	5.2 Preference for simplicity and clinician’s expertise to measure balance challenge

### Theme 1: Influence of task familiarity on perceived balance challenge

Participants indicated that unfamiliar tasks tended to be more challenging for them to maintain balance. The novelty of a given task shaped their perception of balance challenge and the repetition of tasks led to increased familiarity and a subsequent decrease in perceived challenge.

#### Sub-theme 1.1: Challenges of novel tasks.

Many of the voluntary tasks included in the RBT intervention were not commonly performed by participants during daily life, and this was highlighted as a contributing factor to determining whether a task was considered challenging. For example, one participant identified crossover stepping as a task that presented a balance challenge, stating, “The crossover stepping task [was challenging] because it’s also something I usually wouldn’t do.” (RBT10). Other tasks identified as challenging due to unfamiliarity were kicking, side stepping, walking backwards, and leaning backwards during posterior weight shifting. Additionally, participants described that unfamiliar tasks challenged balance because they involved uncommon movement patterns that required activation of different muscle groups. As one participant described, “It makes you use different muscles … you’re side stepping… you’re focusing on one leg and different muscles. It feels different. Haven’t done that in a while.” (RBT13).

#### Sub-theme 1.2: Repetition reduces perceived balance challenge.

Although many tasks were described as unfamiliar and therefore challenging, repetition of these tasks was reported to improve familiarity and reduce perceptions of balance challenge through increased confidence and task performance. Participants reported tasks becoming easier and less challenging over time with more repetition. One participant highlighted “The repetition of it, that’s when...it was getting easier.” (RBT19). Another participant described the change in perceived challenge from the start of the intervention: “It was harder in the first place when [the clinician] started doing it in the first few sessions. It was harder for me to do a lot of activities...but as we passed on to the later sessions, well, I feel I can do them...the ones that were more challenging for me are easier now.” (RBT14). Additionally, participants described gains in confidence through repetition, which led to decreased challenge: “If we did more... I would’ve probably had more confidence … just repetition.” (RBT13).

### Theme 2: Influence of fear of falling on perceived balance challenge

The fear of falling during training tasks was highlighted as a significant factor in the perception of balance challenge intensity. Importantly, a safety harness was worn during training and was found to influence fear of falling and thus the perception of balance challenge.

#### Sub-theme 2.1: Fear as a determinant of high intensity of balance challenge.

A fear of falling or losing balance while performing a task was perceived to increase the level of balance challenge. As one participant described: “I think an activity that makes me scared of falling or it’s harder to perform is a challenging activity for me. Anything that increases the sense of [fear] or when I get scared, that’s mostly challenging for me.” (RBT14). Another participant explained that the risk or likelihood of falling influenced their perception of balance challenge: “A task that would make it more challenging for balance is one that I would feel like I’m going to fall, or that gets me out of balance... [if] I feel like I’m going to fall, that’s a challenging task.” (RBT10). This perception was also reflected in the participants’ rating of the intensity of balance challenge, where tasks that evoked a fear of falling or loss of balance were given higher ratings. As one participant explained: “When you’re looking at the scale, you’d be like, okay, am I going to fall if I do this or am I not going to fall?” (RBT19).

#### Sub-theme 2.2: Emotional and physical safety considerations.

The safety harness worn by participants was reported to reduce fear during balance training tasks and may have influenced the perception of balance challenge. Some participants associated high ratings of balance challenge with scenarios where they would “have fallen if not for the harness.” (RBT07) or relied on it to maintain balance: “If I were leaning hard on the harness, that would be 7, 8.” (RBT18). Another participant described that although they felt safe using the harness, they did not want to rely on it after a fall: “Even though I was safe in the harness, you’re just like, okay, am I going to hang up? Going to twist the wrong way and then get stuck in the harness?” (RBT19). The harness was reported to increase confidence in executing training tasks. As one participant described: “If I didn’t have the harness, I don’t think I would be able to kick with that confidence, having that security of not going to fall.” (RBT10).

### Theme 3: Misuse of the ordinal scale

The 11-point ordinal scale was intended to be used in the study to ensure an adequate level of intensity of balance challenge (i.e., rating of ≥7) was achieved with the exercises prescribed during training. However, participants reported using the scale as a tool to track improvements over time, compare changes in performance, or to moderate the clinician‘s prescription of tasks.

#### Sub-theme 3.1: Tracking personal progress.

The ratings given using the scale were seen as a way of demonstrating increased balance capacity over the course of the training intervention. For example, one participant described the progression of ratings by stating: “When I first started this, I was doing like 6 and 7s, the second week was like 5 and 6s, things started to progress differently, and now we’re at lots of 3s.” (RBT11). Participants reported that progressing from a higher number rating at the start of the training program to a lower number rating at the end indicated that they had improved their balance control. As one participant reflected: “It shows your improvement and progression in the training... it gives you a scale to show and mark and scale your productivity.” (RBT20).

#### Sub-theme 3.2: Comparing performance to pre-injury.

Some participants compared current performance to their abilities before SCI, using the scale to gauge recovery of function and balance capacity. RBT15 indicated that they were basing their ratings on a feeling of confidence doing a similar task before their injury: “I also related it to prior to my accident, to how I felt confident [doing tasks] now compared to then.” They went on to explain their reasoning for higher or lower ratings by stating that “If there is more of a discrepancy between my performance before my injury and after, that’s something that’s more challenging. If there’s less of a discrepancy between performance, then it’s a lesser rating.”

#### Sub-theme 3.3: Controlling the difficulty level of tasks.

Participants described their ability to influence the difficulty level of the tasks by providing artificially low or high ratings to the clinicians who were prescribing the tasks. Some participants believed that individuals seeking a high level of challenge would likely provide low ratings: “If you have the character like me… I would say people of that nature would tend to rate lower.” (RBT18). Alternatively, other participants reported that they deliberately provided higher ratings on the scale to avoid the prescription of more difficult tasks: “I think I probably kind of cheated it because I was more fearful of what might be added if I said it was a zero or a one. So that’s why I never went to the further end of the scale of the very difficult.” (RBT19).

### Theme 4: Ambiguity of ordinal scale

Many participants believed the ordinal scale was confusing and may have led to inconsistent interpretations of the ratings. Participants described being uncertain about the specific meaning of each number on the scale despite being introduced to the scale and using it during each training session.

#### Sub-theme 4.1: The ordinal scale was confusing to use.

The ordinal scale used in training was difficult for participants to use and understand. RBT16 acknowledged this by stating, “Actually, I don’t understand [the ordinal scale] well enough.” Participants described that confusion led to uncertainties using the scale throughout the training interventions. For example, one participant explained: “Well, I found it sort of confusing. I couldn’t use it as a guide from one day to the other.” (RBT17). As another participant described, confusion regarding the scale made it difficult to provide an accurate rating of intensity of balance challenge: “I found it could be a little bit confusing because sometimes it’d be like what number do I give that?” (RBT19).

#### Sub-theme 4.2: Challenges using the extremes of the ordinal scale.

Many of the participants highlighted their challenges with understanding the far extremes of the scales. RBT17 reported having difficulty distinguishing between tasks that had higher levels of intensity of balance challenge: “If it was very difficult, I can’t tell if it’s 8, 9 or 10. I just say it’s difficult.” Other participants described avoiding the use of ratings on either end of the extreme: “I have never had 0 and I have never had 10.” (RBT18). As another participant explained, the confusion associated with the extremes led to them providing ratings in the middle of the scale: “I don’t know what 0 means and I don’t know what 10 means. I can rate myself in the middle somewhere, but I may be at 10 in some of those [tasks]. I don’t know how to push to 10 or 0. I don’t know how to read it. I tend to stay somewhere from 3 to 8.” (RBT16)

### Theme 5: Concern of the time demand of scales

Participants expressed concerns about the time-consuming nature of the scales used to rate the intensity of the balance challenge during training. They were resistant to the addition of the checklist from the BIS-E, and noted the training time lost while completing self-reported assessments. Participants highlighted their priority for active training time and described a preference for a rating scale that is concise and efficient.

#### Sub-theme 5.1: Training time prioritized over rating time.

The objective item checklist included in the BIS-E was criticized for consuming valuable training time. For example, RBT 15 described: “I think [the checklist] would take too much time during the performance of the therapy. You’d spend a lot of time trying to answer questions rather than actually physically doing the exercises.” Another participant felt similarly: “[the checklist] is a little long too…it would cut a lot into the training …you’re spending more time in between exercises.” (RBT10). Participants also described their anticipated frustration with being asked to rate each task: “That would take so much time! [the checklist] would be such a time-consuming thing.” (RBT17).

#### Sub-theme 5.2: Preference for simplicity and clinician’s expertise to measure balance challenge.

When introduced to the items included in the BIS-E checklist that were related to objective markers of balance control, participants expressed a preference for concise tools that clinicians could administer quickly. For example, one participant explained that asking questions based on objective measures “seems much more complicated. It’s a lot more detail-oriented, and it probably answers more questions and gives you more knowledge, but then it comes into a time resolution issue.” (RBT20). Additionally, some participants did not see the value in the information gathered from their ratings. For example, when asked if the participant-rated scales were useful tools, RBT17 stated “Not really, unless it helps [the clinicians], but even then, in my case the answers aren’t necessarily the same as it was the day before.” Many participants valued the clinicians’ expertise over their own perception of balance challenge when adjusting the difficulty of tasks. For example, as RBT17 explained: “The therapist can almost tell if it’s taking much effort or not…they do such a good job and they don’t waste time.”

## Discussion

This study explored the perspectives of individuals with iSCI on measuring the intensity of balance challenge during RBT. Participants rated the perceived intensity of balance challenge during a six-week RBT program using an 11-point ordinal scale, provided verbal explanations for their ratings, and participated in semi-structured interviews. The findings identified five themes: 1) Influence of task familiarity on perceived balance challenge, 2) Influence of fear of falling on perceived balance challenge, 3) Misuse of the ordinal scale, 4) Ambiguity of the ordinal scale, and 5) Concern of the time demand of scales. Collectively, these results suggest that self-reported ratings of balance challenge intensity may be inconsistent and inaccurate. Participants prioritized active training time and valued clinician expertise over their own subjective ratings of balance challenge intensity. As RBT is a specific type of balance training that involves externally applied perturbations and a safety harness, this context should be considered when interpreting and applying the study findings.

The intensity of balance challenge is difficult to conceptualize, and many balance training interventions fail to include measures of intensity [30]. Recently, efforts have been made to define balance intensity. Studies of older adult populations have suggested that, during exercise, balance intensity is dependent on the relationship between the postural control demand of a task and an individual’s maximum capacity to maintain balance during that task [25]. Hence, the factors influencing postural control, as outlined by the Systems Framework for Postural Control, are integral to the concept of balance intensity; for example, sensory integration, cognitive processing and motor systems (e.g., muscle strength, tone) are interacting factors that influence the ability to maintain balance during a task [50,51]. However, these factors were not often mentioned when participants of the current study discussed balance intensity. The conception and understanding of balance challenge for the participants with SCI were shaped by factors such as task novelty and their perceived risk of falling, which may have limited their ability to objectively consider the balance demand of a given task relative to their true maximum capacity. Further, as there is considerable heterogeneity in the sensorimotor deficits experienced by the iSCI population, each individual may experience balance challenge differently. The findings suggest that the subjective interpretation of balance intensity among individuals with iSCI is complex and difficult to measure through participant ratings.

The American College of Sports Medicine has defined high balance intensity as “the highest level of balance-enhancing exercises that can be tolerated without inducing a fall, or near fall” [20]. RBT is designed to be inherently intensive and challenging, involving repeated exposure to unexpected perturbations [47]. These perturbations are meant to challenge balance to the point of near falls, as participants are intentionally perturbed with a force that will elicit a reactive step [47]. The demanding nature of RBT suggests that it is sufficiently challenging by design and may not require additional balance intensity measurement scales. The clinician’s expertise in observing whether perturbations successfully elicit reactive steps and adjusting the force of manual perturbations as a participant’s balance capacity improves may be sufficient for ensuring adequate intensity.

The intended use of the 11-point ordinal scale was to rate the isolated perception of balance challenge intensity experienced during a task. However, this study indicated that participants misused the scale. Instead, it was used to track personal progress or compare current ability to previous ability before injury. This observed misuse aligns with previous research indicating discrepancies between exerciser self-ratings and the ratings of clinicians [37,39]. One study demonstrated that participants often underestimate the intensity of balance exercise compared to clinicians [37]. It has been suggested that the discrepancy between participant and clinician ratings could be explained by the Dunning-Kruger effect [37], which describes the tendency to overestimate one’s abilities and underestimate task difficulty [52,53]. The current study may add to our understanding of this phenomenon as it relates to balance training for people with iSCI. The participants who described using the scale to track performance across time were likely to underestimate the intensity of balance challenge to reflect a perceived and potentially overestimated sense of improvement and ability. The difficulty of the prescribed tasks during RBT sessions was adjusted throughout training as participant capacity increased. Clinicians increased the magnitude of the manual perturbations to elicit reactive steps consistently. Thus, the true intensity of the balance challenge should have remained consistent and would not decrease to reflect improvement or increased capacity.

Participants reported finding the 11-point scale confusing, expressing uncertainty about the specific meaning of each number. They particularly struggled with distinguishing between higher intensity levels (e.g., 8, 9, or 10), often avoiding these extremes and defaulting to ratings in the middle of the scale. This finding aligns with previous work showing that participants struggled to differentiate between specific levels when providing self-reported ratings of balance intensity using an ordinal scale [37]. Participants may better comprehend balance challenge through a simple, binary understanding (i.e., “higher” or “lower” intensity). Additionally, participants in this study expressed significant concern about the time demand of scales, particularly when introduced to the checklist items of the BIS-E. They prioritized active training time over the time spent using self-reported assessment tools, which they preferred to be simple and efficient. Notably, after initial assessment of the BIS scales, it was recommended to exclude the detailed checklist items and only use the simple 5-point ordinal scale of the BIS-E [39]. Conversely, the entirety of the BIS-T was recommended for use by clinicians [39]. This reinforces the ideas proposed by participants in this study, who valued the clinician’s expertise in assessing and adjusting task difficulty over their own subjective ratings. The perceived time cost and inconsistencies in self-reporting may suggest that participant-rated scales, even in simplified versions, may not provide sufficiently accurate or useful supplementary information to justify the interruption of valuable training time. Our findings suggest that individuals with SCI view the clinician’s expert judgment as a more efficient and effective approach to ensuring adequate intensity during RBT.

### Limitations

This study focused on participants who received RBT, which is just one type of balance training for individuals with neurological conditions like iSCI [28,29]. The unique characteristics of RBT, including the unpredictable perturbations, emphasis on reactive stepping and the use of a safety harness [47], could specifically influence participants’ perceptions of challenge and their use of balance intensity measurement tools. The insights gained may be specific to the context of RBT and may not apply to all balance interventions available for individuals with iSCI. Additionally, of the 12 participants included in this study, nine were men and three were women. This gender imbalance limits the ability to make comprehensive conclusions regarding gender-specific experiences or perceptions. For example, fear of falling is a significant concern for individuals with iSCI [3,54], and differences in fear of falling exist between genders [55]. If there are gender-specific differences in how fear of falling influences perceived balance challenge, they may not be reflected in the study’s findings. The sample size was limited by the number of participants who participated in the larger clinical trial [42]; however, a sample of 12 participants was considered to hold sufficient information power to answer the research question. [56] Specifically, the narrow aim of the study, sample specificity, use of established concepts and theories of balance intensity and postural control, and the high quality of dialogue would support a smaller sample size for this study (i.e., 10–12 participants) [56]. Finally, interviewing as a data collection method is inherently at risk of social desirability bias, or the tendency of participants to provide answers aligned with those perceived to be socially acceptable [57]. Several steps were taken to reduce the likelihood of social desirability bias in this study; for example, an interview guide was employed, more than one source of information was collected (i.e., field notes, interviews) to enable data triangulation, and multiple researchers were involved in data analysis [57,58]. Further, the interviewer, who delivered some of the RBT training sessions, was known to participants, and hence, the established rapport and relationship of trust were expected to facilitate honest discussion [57,58].

## Conclusions

Five themes were identified: the influence of task familiarity on perceived balance challenge, the influence of fear of falling on perceived balance challenge, the misuse of the ordinal scale, the ambiguity of the ordinal scale, and concern of the time demand of scales. These perspectives highlight the complexity of assessing balance challenge intensity among individuals with iSCI. Future efforts should consider using quantitative methods to evaluate the validity of self-reported measures of balance intensity for the iSCI population and explore the tools and skills used by SCI clinicians to assess intensity of balance challenge in this population.

## Supporting information

S1 Table Supporting quotes. Additional participant quotes by theme and sub-theme.
(XLSX)
